# Moving from Theory to Practice: Key Concepts and Operational Factors to Transform Surveillance as a Core Intervention

**DOI:** 10.4269/ajtmh.23-0019

**Published:** 2023-01-23

**Authors:** Arnaud Le Menach

**Affiliations:** Analytics Surveillance, and Technology, Clinton Health Access Initiative, Boston, Massachusetts

The World Health Organization (WHO) defines surveillance as a core intervention for malaria, naming it one of three pillars in its Global Technical Strategy (GTS) for Malaria 2016–2030.^1^ A strong surveillance system is critical for an efficient and effective malaria program, supporting strategy planning, guiding its operationalization, directing resources, targeting interventions to the right places and populations, monitoring disease trends, and addressing any programmatic gaps identified during the evaluation of program performance. As the level of malaria transmission reduces and cases are reported individually and more frequently at a higher spatial resolution, surveillance information should further trigger a locally tailored response in every remaining area of sustained transmission.^2^

To systematically track national progress towards achieving Pillar 3 of the GTS, WHO recommends the regular monitoring and evaluation of surveillance systems. In collaboration with the Clinton Health Access Initiative and partners, WHO developed a toolkit that focuses on two components. The first component involves the evaluation of surveillance system performance, including assessing the quality of generated data, its representativeness, and its use for decision-making. The second component consists of the drivers of performance such as infrastructural aspects, processes associated with the flow of information from data collection to response, and behavioral aspects of staff involved in surveillance.^3^ These types of information are valuable to identify key gaps and inform surveillance systems improvements.

While there has been progress in promoting the use of digital solutions and supporting the rollout of integrated data platforms, several factors are preventing surveillance systems from effectively guiding decision-making and monitoring progress accurately. For example, in Sub-Saharan Africa the quality of surveillance data still does not permit a robust estimate of the burden of malaria cases in the World Malaria Report, and estimations continue to rely on modeled data from household prevalence surveys.^4^ Several drivers can explain these shortcomings, such as: a lack of information reported from the private sector or communities; the fragmented nature of information systems where case, commodity, entomology, or interventions data sit in different databases managed by different stakeholders; limited access to timely information and evidence at different levels of the health system, especially at the local level; suboptimal staff capacity to review, analyze, and interpret data; and a lack of resources.

Furthermore, progress towards strengthening health systems has been hampered by the COVID-19 pandemic and its global impact on every endemic country, bringing disruption to the delivery of commodities such as bednets or diagnostic tests and the diversion of resources from diseases such as malaria. However, not all the impacts of COVID-19 have been negative. Most countries already had systems in place that were leveraged to monitor a set of disease indicators throughout the pandemic and to help countries to better understand whether malaria case management services continued to operate, or if disruptions to service provision or care seeking occurred. In addition, the renewed focus on surveillance information to monitor COVID-19 trends provided an opportunity to strengthen routine health information systems across various diseases, including malaria.

The tasks at hand to improve surveillance may seem daunting at first but focusing on a few key priorities around improving the quality (such as the concordance of data between registers and electronic reports) and use of information will enable these systems to generate high-quality data that can be trusted by programs and partners and can effectively guide countries in their efforts to reduce malaria burden and stop transmission.

The articles in this supplemental issue provide insight into how to address surveillance gaps and present key concepts to transform current surveillance systems into a core intervention for malaria. A strong country leadership will be required to achieve the goal of having country-owned, high-performing surveillance processes with high-quality data that are reported in a timely manner, represent all sectors (public, private, and community), can be accessed at all levels of the health system through user friendly dashboards, can be reviewed routinely, and can be translated into actions. A high performing malaria surveillance system will rely on effective, sustainable, and context-appropriate digital health information systems, and on human resources and capacity that meet programmatic and community needs, in an environment where the program has the resources to guide system improvements.

Beyond the conceptual framework for developing and maintaining effective surveillance systems, the supplement articles present case studies that outline the need to remain innovative to measure malaria burden, intervention coverage, and impacts of interventions, leveraging other routine systems such as antenatal care, which provide a complementary approach to other sources of information from routine public health facilities or from household surveys. Finally, the supplement provides a reminder that in an ever-evolving malaria epidemiological context, surveillance can be used to monitor and measure emerging trends, such as quantifying the importance of the private sector in the Greater Mekong Sub-region or describing the increase of urbanization in malaria endemic areas and the characteristics of urban malaria cases in Mozambique.

An enabling environment for malaria surveillance improvements will rely on government leadership, vision, commitment, coordination, and regulation. A strong governance structure and strategic policy will be paramount for efficient, effective, and equitable surveillance program planning and implementation, along with sustainable human resources and funding.
